# On the Mechanics of Immediate Corrections and Aftereffects in Prism Adaptation

**DOI:** 10.3390/vision1040027

**Published:** 2017-12-19

**Authors:** Klaudia Pochopien, Karoline Spang, Torsten Stemmler, Manfred Fahle

**Affiliations:** 1 Department of Human-Neurobiology, University of Bremen, Hochschulring 18, D-28359 Bremen, Germany; 2 Gierather Straße 195 A, D-51469 Bergisch Gladbach, Germany

**Keywords:** prism adaptation, immediate correction effect, direct effect, perceptual learning, psychophysics

## Abstract

Prisms laterally shifting the perceived visual world cause arm movements to deviate from intended targets. The resulting error—the direct effect—both for pointing and throwing movements, usually corresponds to only around half of the prism’s optical power due to an “immediate correction effect”. We investigated the mechanisms of this immediate correction effect. In three experiments with 73 healthy subjects we find that the immediate correction effect is associated with a head and/or eye rotation. Since these rotations are subconscious they are not taken into account by the participants. These subconscious rotations compensate for a large portion of the prism’s optical effect and change the subjective straight ahead. These movements seem to be induced only in a rich visual environment and hence do not take place in the dark. They correspond to the difference between the direct effect and the optical power of the prisms and seem to cause the immediate correction effect. Hence, eye-hand adaptation only adapts to the prism’s optical power minus unconscious head rotation and hence is much smaller than the optical power of the prisms.

## 1. Introduction

Eye-hand coordination is essential for daily interactions with the environment, for example reaching towards an object, using tools, opening the door or grasping for a cup of coffee [1]. This eye-hand coordination relies on one of the most important and complex sensory-motor systems in the human body [2]. To produce a correct motor command, signals from the visual system regarding the location of objects as well as information from the proprioceptive system regarding arm/hand position are required [1,2]. The sensory-motor system quasi-automatically adjusts to many changes, for example when using tools or wearing prisms [1,3,4,5,6,7]. 

Prism glasses shift the visual information in the direction of the prismatic shift [1,7,8] while arm/hand proprioception stays unchanged. This leads to a discrepancy between the seen versus felt position of the arm and to mispointing in the direction of the prismatic shift (direct effect), which is consciously perceived during movements. The initial mislocalization induces adaptive changes during repeated movements during unrestricted view of the movements (adaptation) [9,10,11,12,13]. After removal of the prism glasses, a pointing error in the direction opposite to the prismatic effect appears (aftereffect) [3,7,14]. The pointing error disappears gradually within a few movements without prisms (readaptation).

Horizontally shifting prisms induce initial mispointing that amounts, however, to only half the size expected on the basis of the prism’s optical power, due to an immediate correction effect [14,15,16,17]. Earlier, we found that in the dark the prism effect roughly corresponds to the optical power of the prisms, that is, the immediate correction effect disappears for pointing movements [18]. 

In three experiments, we tested the relation between the immediate correction effect and subconscious head, eye and trunk rotation. Our hypotheses were (a) these rotations only occur in a rich visual environment, i.e., with lights on and (b) that the combined size of these movements corresponds quantitatively to the size of the immediate correction effect.

## 2. Results

### 2.1. Experiment 1: Pointing and Throwing

**Figure 1:**
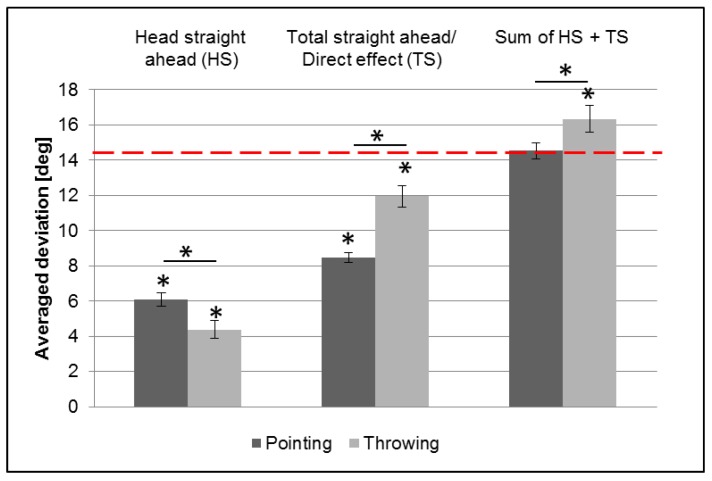
*Head* straight ahead, *direct* effect and sum of head straight ahead and direct effect after adaptive interval (before adaptive movements) in light (first interval test). The red dashed line on the y-axis indicates the optical power of the prisms. Error bars indicate SEs. * indicates *p* ≤ 0.05. Head rotation is larger for pointing, associated with a smaller direct effect caused by a larger immediate correction effect.

Our main hypothesis was that the size of the immediate correction effect corresponds quantitatively to the size of immediate and unconscious changes in *head* position. To test this hypothesis, we measured subjective straight ahead of the *head* before and while wearing prisms. Figure 1 shows the averaged results of the felt *head* straight ahead position (i.e., orienting the head in the direction of the body midline with closed eyes), the felt arm straight ahead (i.e., positioning the hand in the direction of a central target without visual control despite of open eyes) for pointing and throwing (hitherto called: *total* straight ahead), as well as the sum of both conditions. Measurements were taken after an adaptive interval—that is with prisms on but before any feedback regarding felt versus seen hand position. We averaged the deviations of the right-shifting prisms group with the inverted deviations of the left-shifting one, since they did not differ significantly from each other (unpaired *t*-test). For both conditions, the results of felt *head* straight ahead (HS; pointing: *t* = 16.1; *p* < 0.001; throwing: *t* = 8.7; *p* = < 0.001) and *total* straight ahead (TS; pointing towards a central *target* without visual feedback; pointing: *t* = 31.3; *p* < 0.001; throwing: *t* = 20.2; *p* < 0.001) deviate significantly from zero in the direction of the prismatic shift (one-sided *t*-tests), that is, we find significant prism adaptation even before any arm movements. As a result, aiming at a visual target deviates from that target when participants wear prisms, significantly less than the optical power of the prisms (pointing: *t* = −21.3; *p* < 0.001; throwing: *t* = −3.8; *p* = 0.001).

Results differ between pointing and throwing (two-sided paired *t*-test) for both felt *head* straight ahead and *total* straight ahead. Head rotation was significantly larger for pointing than for throwing (*t* = 2.4, *p* = 0.02) (Figure 1). This means that to fixate the target, eyes had to be in a more eccentric position in the orbit during throwing than during pointing. In analogy, the direct effect, that is the first movement under prisms, was significantly larger for throwing than for pointing (*t* = −5.4, *p* < 0.001), suggesting a relation between head rotation on one side and the size of the immediate correction effect on the other side.

The results for the subjective *total* straight ahead (TS) as measured by aiming at the target (the first measurement of which is called the direct effect) here represent the difference between the optical power of the prisms and of immediate and unconscious head and gaze changes when putting on prisms but before visual feedback on hand position. 

In other words: the sum of the direct effect (mislocation of target: TS) and the subjective *head* straight ahead (unconscious head rotation: HS) corresponds to the optical power of the prisms and is for throwing even slightly larger than the optical power (Table 1 and Figure 1). Hence the amount of head rotation corresponds well to the immediate correction effect, in line with our main hypothesis. The remaining small difference between the sum of unconscious head rotation (HS) and the *total* straight ahead (TS) on one hand and the optic power of the prisms is not significant for pointing and even larger than the optical effect for throwing (Table 1). The sum of the subjective *head* straight ahead and the direct effect (HS + TS) differed significantly (*t* = −2.1; *p* = 0.048; two-sided paired *t*-test) between pointing and throwing. 

### 2.2. Experiment 2: Central versus Rotated Chair in the Dark

Even in the dark with hardly any unconscious head rotation, the direct effect (DE) is slightly smaller than the prism’s optical power [18]. We investigated the reason for this small remaining deviation of data from our main hypothesis. Our expectation was that this remaining factor consists mainly of a bias against large eccentric arm movements. In this experiment, therefore, we rotated the participant’s body in the direction of the prism shift, so they performed less eccentric movements to reach the perceived target.

#### 2.2.1. Direct Effect: Central versus Rotated Chair Position

**Figure 2:**
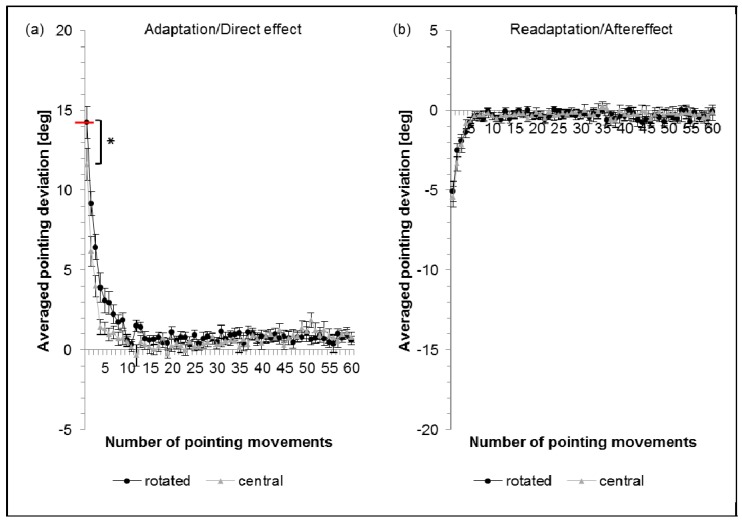
(**a**) Prism adaptation and direct effect; (**b**) Readaptation and aftereffect. Black dots show the results for the rotated chair position and grey triangles the averaged results for the central chair position. Error bars denote SEs. Y-axis: horizontal deviation of the averaged pointing movements from the central target (y = 0), the red bar indicates the optical power of the prisms. X-axis: number of pointing movements performed. * with bar indicates a significant difference (*p* ≤ 0.05) of the first movement between central and rotated chair condition, hence a difference in the immediate correction effect. As is normal in prism experiments, the aftereffect is much smaller than the direct effect, probably due to the fact that the unconscious head rotation disappears when the prisms are removed.

Figure 2a shows the averaged pointing errors during adaptation. For the rotated chair condition in darkness the direct effect closely matched the optical shift of the prisms (100%) (difference not significant) while with central chair condition the direct effect amounted to only 82% (significantly different from 100%) (Table 2) in line with previous results [18]. As a consequence of this smaller immediate correction effect, the initial pointing error was significantly larger by 2.7° for the rotated chair condition than for the central chair condition (two-sided paired *t*-test). 

#### 2.2.2. Aftereffect: Central versus Rotated Chair Position 

Figure 2b shows the averaged pointing errors during readaptation. Both conditions (central and rotated chair) produced a significant aftereffect (AE) in the direction opposite to the prismatic shift (central: 38%; rotated: 36%) which decreased with subsequent pointing movements. The aftereffect did not differ significantly between the central and rotated condition.

#### 2.2.3. Adaptive Components: Central versus Rotated Chair Position

**Figure 3:**
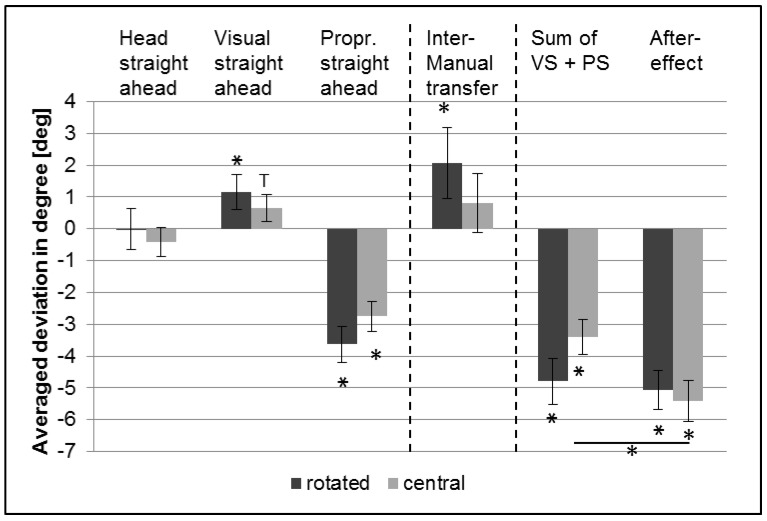
Adaptive components after prism adaptation in the dark (aftereffects) without prisms. Dark bars: adaptation with rotated chair; light bars: adaptation with central chair. Averaged group results for subjective *head* straight ahead, *visual* straight ahead (gaze direction), *proprioceptive* straight ahead and intermanual transfer as well as the sum of *visual* and *proprioceptive* straight ahead and aftereffect. Error bars indicate SEs. * indicates *p* ≤ 0.05, T = Trend. There was no intermanual transfer since movements deviated in the direction opposite to that of the adapted arm.

Figure 3 shows the averaged results of subjective head, visual and right arm straight ahead direction for all 28 participants of the second experiment as well as the results for the left arm position for 14 participants after adaptation (first posttest), for both rotated and central chair position after removing the prisms (aftereffects). Moreover, the sum of the *visual* and the *proprioceptive* straight ahead, as well as the aftereffect are shown.

In the dark, subjective straight ahead of the *head* did not change significantly (one-sided *t*-test). But the subjective *visual* straight ahead (VS; gaze direction) adapted significantly in the direction of the prismatic shift for rotated chair position (*t* = 2.1, *p* = 0.02), whereas in the central chair position (*t* = 1.6, *p* = 0.06) we found only a trend (Figure 3).

The most pronounced significant adaptation occurred for felt, i.e., proprioceptive *arm* position (PS: *proprioceptive straight* ahead; right arm) under both chair conditions (rotated: *t* = −6.3, *p* < 0.001; central: *t* = −5.9, *p* < 0.001) (Figure 3). 

The subjective left arm position deviated significantly to the right (i.e., in the “wrong” direction) in the rotated condition (*t* = 1.9, *p* = 0.04) while not in the central condition, indicating a complete lack of intermanual transfer (IT) in the expected direction. In the dark, there is hardly any *visual* adaptation but mostly adaptation of the arm/hand motor system. We attribute this apparent effect of intermanual transfer to the (small) change in (perceived) *visual* straight ahead since this adaptation affects both arms.

For both conditions, the sum of the *visual* (results were inverted) and the *proprioceptive* straight deviated significantly in direction against the prismatic shift (rotated: *t* = −6.7, *p* < 0.001; central: *t* = −6.2, *p* < 0.001). The same was true for the aftereffect (rotated: *t* = −8.2, *p* < 0.001; central: *t* = −8.4, *p* < 0.001). Two-sided paired *t*-tests indicate no significant differences between the adaptive components for the rotated versus central condition.

The sum of changes in subjective *visual* straight ahead (gaze direction) and *proprioceptive* straight ahead—that is the adaptive changes in the felt straight ahead directions for gaze and for pointing—corresponds relatively well to the size of the aftereffect (difference significant only for the central position; *t* = 2.7, *p* =0.013; two-sided paired *t*-tests) (Figure 3; Table 3). These results indicate that trunk rotation in the direction of the prismatic shift by means of rotation of the chair leads, in the dark, in the absence of any unconscious head rotation to mispointing (a direct effect) corresponding to the prism’s optical power.

### 2.3. Experiment 3: Light versus Dark Conditions

To more closely investigate the dependence of the direct effect on subconscious hand and gaze rotation, we compared the direct effect, the aftereffect as well as the subjective *visual* straight ahead for two different light conditions that yield different amounts of immediate prism correction.

#### 2.3.1. Direct Effect

**Figure 4:**
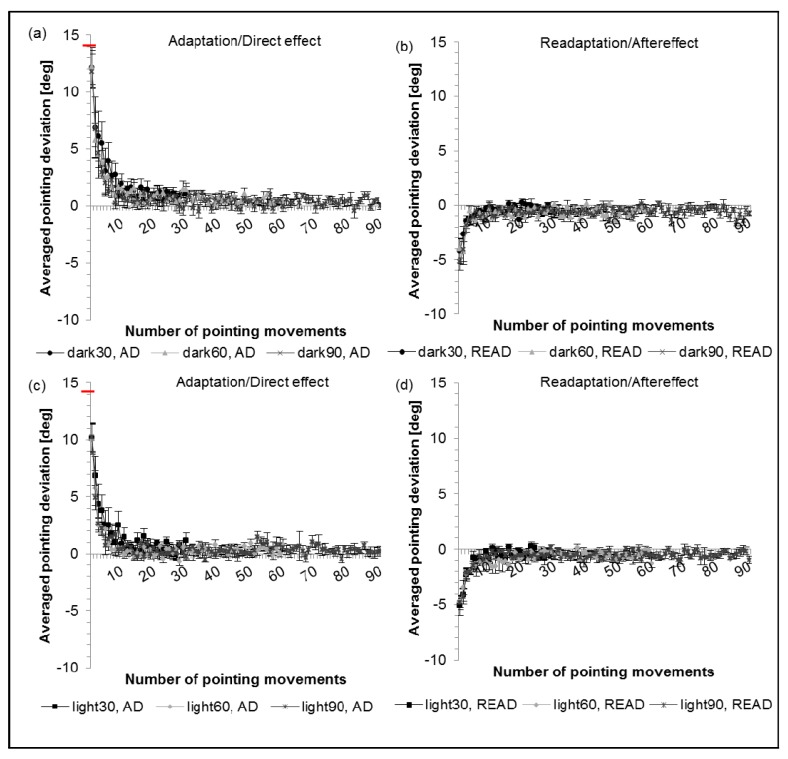
(**a**,**c**) Prism adaptation and direct effect; (**b**,**d**) Readaptation and aftereffect. Dots/triangles/crosses show the averaged results for 30/60/90 pointing movements, respectively, in the dark condition and squares/rhombus/stars the averaged results for 30/60/90 pointing movements in the light condition. Error bars denote SEs. Y-axis: horizontal deviation of the averaged pointing movements from the central target (y = 0), the red bar indicates the optical power of the prisms. X-axis: number of pointing movements performed.

Figure 4 shows averaged results for 30, 60 and 90 pointing movements performed in the dark (Figure 4a) as well as light conditions (Figure 4c). Similar initial pointing errors (direct effects) in the direction of the prismatic shift and around the size of the prismatic shift (14.2°) emerge for all three session lengths in the dark. As in Experiment 2 (Table 2) the direct effects in the dark almost reach the actual prismatic power (83% to 86%). In the light, however, the initial pointing error is significantly smaller than 100% for all conditions (71% to 72%) (Table 4). 

#### 2.3.2. Aftereffect

Figure 4 depicts the initial pointing errors during readaptation for both conditions (dark and light) for 30, 60 and 90 pointing movements. In the dark (Figure 4b) as well as in the light condition (Figure 4d), the aftereffect differed significantly from zero (one-sided *t*-test) and decreased with further pointing movements. In the dark, the aftereffects were between −28% and −37%, while between −34% and −36% in the light.

## 3. Discussion

Several components can contribute to prism adaptation, especially changes in perceived arm position, perceived head position and perceived eye position [4,9,19,20,21,22]. 

To assess the influence of prisms in our experiments, one has to keep in mind that the optical shift of the prisms is immediately compensated for by eye movements that ensure that the visual target’s image falls still on the foveolar. This means that the eyes are at an eccentric position which is usually mildly disagreeable. Hence, participants tend to rotate their head or else their whole body (if possible, i.e., under the rotated chair position) to decrease eccentric eye position.

### 3.1. Test of First Hypothesis: No Head Rotation in the Dark 

We investigated the mechanisms underlying the immediate correction effect, i.e., the fact that initial mispointing and misthrowing—the direct effect—of participants wearing prisms is only about half the size to be expected on the basis of the prism’s optical power [14,15,16,17]. Based on a previous investigation that found the immediate correction effect to disappear in the dark [18], we supposed that the immediate correction effect relies on some (subconscious) spatial memory components of participants about the structure of the room and their own body position that may trigger postural adjustments to a shifted visual environment when wearing prisms. More precisely, people know that the room has not moved while they put on the prisms, so brains have good reasons to attribute the shift of the visual surround on the retina to an “overlooked” eye or head movement. Our hypothesis was that when this spatial room information is removed in a dark room, the immediate correction effect (almost completely) disappears and the mispointing corresponds to the optical power of the prisms [18].

Our more specific hypothesis tested here, based on informal observations of participants and some hints in the literature, was that when wearing prisms, participants perform subconscious head and eye rotations to partly compensate the prismatic shift. Previous informal reports on changes of head rotation and/or of changes in subjective *visual* straight ahead can be found in a number of older papers but were not addressed by these authors [20,23]. However, we are not aware of any measurements correlating head orientation with the immediate correction effect.

The lack of immediate correction effect in the dark condition suggests that this subconscious body adjustment might not occur in the dark and this is the reason why the immediate correction effect disappears. Again, from informal observations we expected that throwing under prisms leads to a smaller head rotation than for pointing. Then, the immediate correction effect should be smaller and hence the direct effect should be larger. The first experiment therefore measured head position for both pointing and throwing under prisms. These conditions indeed differ in the amount of subconscious head rotation and we find a subconscious head rotation corresponding in size roughly to the immediate correction effect, larger for pointing than for throwing.

In the second experiment, we measured eye direction (indirectly, by gaze direction) as well as head direction before and after wearing prisms in a dark room. Contrary to the first experiment, participants were allowed to rotate their chair and hence their body position as they preferred in one part of the experiment. Head and eye directions did not (subjective *head* straight ahead) or only marginally (subjective *visual* straight ahead as measured by gaze direction) change when donning prisms in the dark as opposed to the results in bright light (compare Figure 1 and Figure 3 but note different scales) in accordance with our hypothesis. The subjective *proprioceptive* straight ahead (measured by pointing to a target with the hand/arm occluded) of the adapted arm showed a significant adaptation over the course of the experiment, while the contralateral arm did not and even deviated in the opposite direction from the adapted arm (see Figure 3) probably as a result of the (small) shift in *visual* straight ahead. These results emerged both for the central and the rotated chair position.

We conclude that in the dark, no substantial subconscious rotation of the eyes and head occurs; and that therefore the immediate correction effect is absent. Adaptation to the prisms under these conditions is to a large extent in the proprioception of the arm, producing a significant change in perceived arm position, only for the arm used during adaptation. This finding argues against a general shift in the representation of space—such a shift should generalize to the unadapted arm.

### 3.2. Test of Second Hypothesis: Unconscious Body Rotations Correspond Quantitatively to the Immediate Correction Effect

In the third experiment, in line with our previous finding in the dark, we again found no immediate correction effect, while the immediate correction effect was present with the lights on.

This finding might be caused by the fact that the chair (and hence body) was rotated in the direction of the prismatic shift and that this rotation was not taken (fully) into account by the participants. The chair was rotated by around 5 to 9 degrees in the direction of the prismatic shift, thus reaching the amount of head and eye rotation that we observe for head rotation in the light and which makes foveation of the target easier with prisms on. Moreover, head rotation (in the bright condition) decreases the target’s initial eccentricity on the retina—the further the head is rotated, the less the eye has to be moved in the orbit to fixate the target.

In any case, head or body rotation in the direction of the prism’s optical shift decreases the required eye movements to fixate the target. Body rotation in direction of the prism deviation makes further head rotations apparently redundant. It fits well with this argument that subjective ‘straight ahead’ of the *head* in the second experiment was at zero rotation (Figure 3).

We find that, in line with our second (main) hypothesis, the amount of head rotation corresponds well to the size of the immediate correction effect; i.e., to the difference between optical power of the prisms and the size of the direct effect.

In conclusion, our data support both of our hypotheses outlined in the Introduction. Unconscious head rotation in the direction of the prismatic effect occurs only when observers have visual information about their surroundings and hence may attribute at least part of the visual shift produced by the prisms to a missing reference copy to the eyes or head. The unconscious head rotation—that is missing in the dark—corresponds well to the size of the immediate correction effect, that is to the difference between the optical power of the prisms and the amount of initial mispointing (the direct effect). Hence, we argue that almost half of prisms adaptation is due to an unconscious rotation of the head/eye system. 

## 4. Materials and Methods 

### 4.1. Ethics Statement

The study was approved by the local ethics committee of the University of Bremen. Participants were informed regarding the aim and the procedure of the study, were treated in accordance with the Declaration of Helsinki (2008) and signed a written consent prior to the study. Participants were paid for participation and were free to withdraw from the study at any time.

### 4.2. Participants and General Procedure

Seventy-three participants, aged between 18 and 30 years (49 female, 24 male; M = 23.8, SE = 0.43), took part in three experiments. All were employees or students of the University of Bremen. Inclusion criteria of participants were: right-handedness, normal or corrected-to-normal visual acuity (20/20; only contact lenses allowed) (Freiburger Visual Acuity Test; [24]), normal stereopsis (Lang Stereo Test; [25]), no previous exposure to prism adaptation and pupillary distance between 54 and 64 mm (Auto-Refractometer; NIDEK ARK-700, Gamagori, Japan). In addition, eye dominance was tested to compensate the direction of deviation from cyclopean view when calculating perceived target directions. 

Experiment 1 tested altogether 37 participants (19 female, 18 male; M = 24.2 years, SE = 0.77; 25 participants with right prisms, 12 participants with left prisms) in an experiment on pointing respectively throwing (two sub experiments) in the light. Experiment 2 tested one group of 28 participants (23 females, 5 males; M = 22.9 years, SE = 0.54) in a darkened room. Experiment 3 tested eight additional participants (7 females, 1 male; M = 25.3 years, SE = 1.24) under two different room light conditions: dark and light.

### 4.3. Experiment 1: Pointing and Throwing

#### 4.3.1. Experimental Set-Up for Pointing

**Figure 5:**
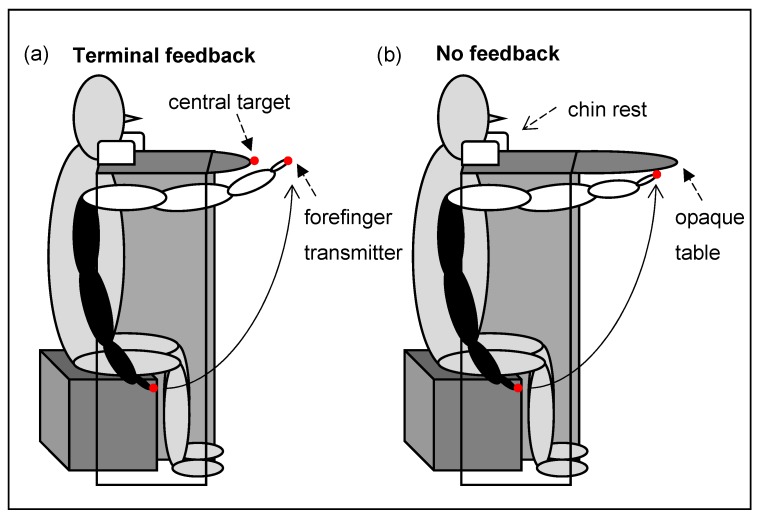
Participant executing a pointing movement towards (**a**) the central target with terminal feedback; (**b**) straight ahead without feedback. The black arm describes the starting position, the white arm the movement endpoint. The red dots depict the central target and the finger transmitter.

In the pointing part of Experiment 1, participants sat at a table 99.5 cm high, 110 cm wide and 57 cm deep (shortened table) (Figure 5a) or 76 cm deep (extended table) (Figure 5b) with their arm underneath the opaque table. A chin rest kept head-target distance at 510 mm. During terminal feedback, only around 4 cm of the index finger was visible at the end of each pointing movement (Figure 5a), while the finger was never seen in the “no feedback” condition (Figure 5b). A red target was mounted on the central target (target transmitter). Miniature ultrasound transmitters (Zebris System) allowed to track the position and the trajectory of the index finger.

**Figure 6:**
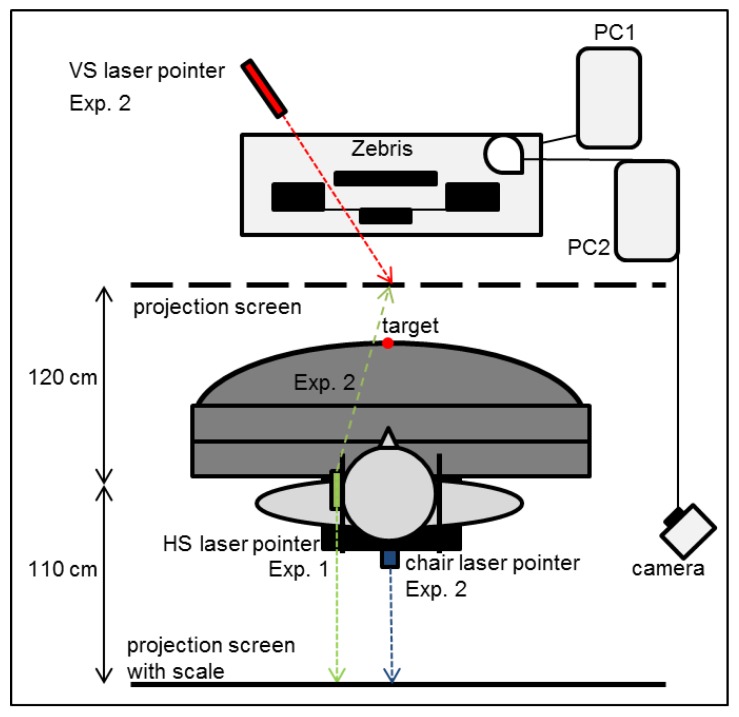
Test set-up from above. A laser pointer (blue; Exp. 2) mounted on the chair projects to a projection screen with measuring scale (black line) behind the participant. White projection screens behind (black line; Exp. 1) and in front of (dashed line; Exp. 2) the participant receive beams of the head laser pointer (green) and of the *visual* straight ahead movable laser pointer (red; Exp. 2), respectively.

Participants wore a forehead strap with a laser pointer whose beam indicated the head rotation and was used to measure the straight-ahead position of the head (HS) on a projection screen behind the participants, recorded by a digital camera (Figure 6). This strap and the chin rest did not restrict observer’s head rotation but just keep viewing distance constant. Participants were tested with either right-shifting prisms (Carl Zeiss, Oberkochen, Germany) with a circular shape (Ø = 35 mm) and an optical center distance of 59 mm, or else with left-shifting prisms with an oval shape (Ø = 45 mm) and an optical center distance of 64 mm. The optical power of both prisms was 14.2° and all participants participated both in the throwing and the pointing experiment.

#### 4.3.2. Procedure for Pointing

Participants were donned the prisms and asked to look around in the laboratory for two minutes, which we call the adaptive interval. Both subjective *head* straight ahead (HS) and subjective *total* straight ahead (TS; that is the direct effect) were measured before (pretest) and after the adaptive interval (first interval test).

To measure the subjective *head* straight ahead, participants were instructed to freely align their felt head position with their shoulders (eyes closed and lights off). The forehead strap laser pointer then indicated head rotation angle (two readings) [17].

To measure the direct effect, the table was extended and lights were switched on. Participants were asked to perform rhythmic movements at a frequency of 0.37 Hz (~2.7 s per movement) towards the visual target and performed two pointing movements with open eyes towards the central target without visual feedback [17,22,26].

#### 4.3.3. Experimental Set-Up & Procedure for Throwing

**Figure 7:**
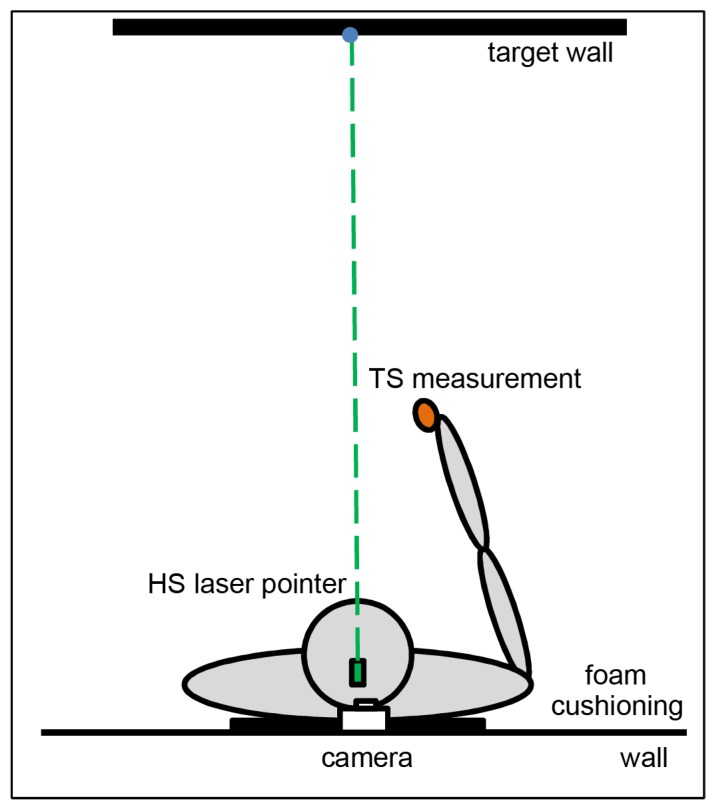
Test set-up for throwing. The participant stood at a wall (black line) with the back at a foam cushioning. The blue dot indicates the central target position (0°). The target wall received beams of the head laser pointer (green) as well as balls for measuring the *total* straight ahead (orange).

In the throwing part of Experiment 1 participants had to throw softballs (24 g, 5.0 cm diameter) towards a visual target on a 1.5 m × 1.5 m wide wall to measure the subjective *total* straight ahead (Figure 7). The target was a black spot (2.0 cm diameter) attached to the wall at a height of 156 cm. Participants stood upright with their mid-sagittal plane aligned with the wall at a viewing distance of 200 cm. With unrestrained head, subjects saw the target under daylight illumination. The wall was layered with Velcro material and the softball adhered to the wall after each throw. A laser pointer attached centrally on the participants’ head was directed towards the wall to measure the subjective *head* straight ahead but switched on only shortly to measure head position. Data were recorded by a digital camera. The prisms employed and the sequence of testing matched that of the previous experiment.

### 4.4. Set-Up & Procedure for Experiment 2: Central versus Rotated Chair in the Dark 

#### 4.4.1. Experimental Set-Up 

**Figure 8:**
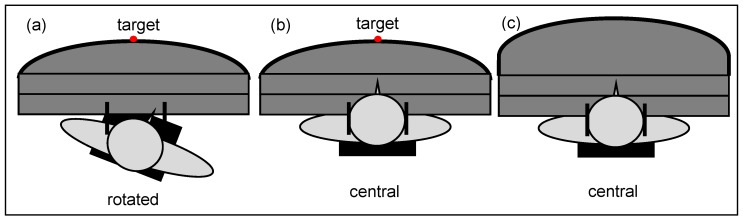
Chair positions: (**a**) prism shift and chair rotation to the right with terminal feedback; (**b**) prism shift to the right and central chair position with terminal feedback; (**c**) central chair with the table extended, hence no visual feedback.

Two chair positions were employed rather than only the central one as in Experiment 1: (a) central at 0°; and (b) chair rotated rightwards individually until the participants felt that the target was straight in front of them while wearing right-shifting prisms (with an optical power of 14.2°). Rotation was on average 8.7° (SE = 0.28). A diode attached on the central target and a second diode plus a transmitter at the index finger provided *visual* feedback in the dark (Figure 5 and Figure 8). A laser pointer mounted on the back side of the chair pointed towards a measuring scale located behind the participants, a second laser pointer attached to the head pointed towards the projection screen in front of the table. They allowed to measure both the chair rotation as well as the direction of gaze (Figure 6).

#### 4.4.2. Procedure

During Experiment 2 the chair was either rotated rightwards or else remained central (0°) (Figure 8a,b). We measured subjective *visual* straight ahead (VS), i.e., gaze direction, subjective *head* straight ahead, subjective *proprioceptive* straight ahead for the right arm (PS) and in a subgroup of 14 observers also for the left arm (intermanual transfer; IT) without feedback and with central chair (Figure 8c) (Table S1 in Supplementary Materials).

To test *visual* straight ahead that measured gaze direction, participants had to indicate when the spot of a laser pointer, starting at a lateral position on the projection screen and moving horizontally appeared to be located exactly straight ahead (i.e., in the center of their visual field), in the dark and without wearing prism glasses [17,22,26]. The head was positioned straight ahead in the chin rest (to keep the distance between participant and target, without any head fixation). Each trial consisted of five movements, with endpoints documented by a digital photo.

The *proprioceptive* straight ahead tested adaptation of the hand/arm system and was measured with the table extended (no visual feedback) and the participant’s eyes closed. With their head in the chin rest participants performed five slow pointing movements to their subjective straight-ahead position (median line of the body) with the right arm (or else with the left arm for intermanual transfer) [17,22,26]. Note that observers performed the same type of movements as with the shorter table, the only difference being that they do not perceive their fingertip at the movement’s endpoint with the extended table.

During adaptation and readaptation, participants performed up to 60 rhythmic pointing movements in the dark at a frequency of around 0.37 Hz (~2.7 s per movement) towards the red central target (0°) and back. Participant were tested twice (rotated right chair and central chair) in counterbalanced order.

After adaptation, the prism glasses were removed and the first posttests were performed. 

During readaptation without prisms, the chair and head positions stayed as in the corresponding adaptation tasks. 

### 4.5. Set-Up *&* Procedure for Experiment 3: Light versus Dark Conditions

#### Experimental Set-Up and Procedure

The experimental set-up (Figure 6) changed relative to Experiment 2 in the following way: Participants were tested twice, counterbalanced between the dark and light conditions, with terminal feedback and always with chair position rotated right by on average 8.4° (SE = 0.20) in dark and 5.7° (SE = 0.22) in light conditions (Figure 6a and Figure 8a). Adaptation and readaptation involved either 30, 60 or 90 pointing movements (Table S2 in Supplementary Materials).

### 4.6. Analysis

Individual baseline measurements (pretest) were subtracted from the corresponding measurement after the adaptation (first interval test/first posttest) for all data where applicable. Positive values indicate a shift in the direction of the prismatic shift; negative values a deviation in the direction against the prismatic shift during the adaptive interval.

#### Data Analysis Pointing

The Zebris system (Zebris Medical, Isny, Germany) recorded the trajectory of the pointing movements in three dimensions and a Matlab program developed in-house determined the first extremum of the finger’s trajectory. Another Matlab program analyzed the position of the laser spots, correcting for optical distortions caused by the fact that eye and camera saw the screen from different angles. Results were verified manually by the experimenter and *t*-tests were employed.

## 5. Conclusions 

In conclusion, our results corroborate the finding that under appropriate conditions in the dark, the direct prism effect corresponds to the optical power of the prisms. The immediate correction effect seems to rely largely on a head and eye rotation in the direction of the prism shift, under normal (light) conditions and more so for pointing than for throwing. The amount of head rotation is smaller for throwing than for pointing, reflecting for example the difference between these types of movements, between the representation of near versus far space and the fact that for throwing observers leaned their back against the wall that provided an extra external system of reference. It is important to note that this head rotation must be unconscious. Since any conscious head rotation does, of course, not change our perception of the visual world. But if observers perform a saccade towards the target after prisms have been donned, the size of this saccade may not be completely taken into account when the brain estimates the positions of visual objects. After all, observers know that the room has not been rotated when they put on the prisms. A similar misjudgment of pursuit eye movements is well known in the Filehne illusion [27]. 

This head rotation reduces the retinal eccentricity of the displaced target (for stationary eye position) and requires a smaller saccade towards the target. Participants seem not to take into account the (full amount of) head and eye rotation towards the target and thus produce the immediate correction effect. In the dark, this head rotation is greatly reduced and the immediate correction effect disappears. We conclude that the head is rotated only if prisms shift a rich visual environment—participants may misinterpret this shift as a head/body rotation. In the dark, however only the target moves and this seems to be interpreted correctly as a mere target movement, not a body rotation.

In all conditions tested, the sum of head rotation and the direct effect corresponded closely to the optical shift of the prisms. In the dark, adaptation to the prisms was more in the arm/hand proprioceptive system but did not transfer between arms. Our results show that the sum of subconscious head rotation plus the direct effect indeed are very close to the optical power of the prisms and that the sum of adaptation for the subjective *visual* straight ahead and the *proprioceptive* straight ahead after adaptation correspond to the aftereffect measured after removing the prisms. So, we here propose possible “mechanics” both for the immediate correction effect and the size of the aftereffect, based on purely unconscious postural adjustment.

## Figures and Tables

**Table 1:** Results and statistical comparison of the sum of unconscious *head* rotation (HS) plus *total* straight ahead/direct effect (TS), for pointing and throwing, after adaptive interval in a bright laboratory, before any feedback on hand position but with prisms on. Two-sided *t*-test against the prism’s optical power.

Condition	Mean (°)	SE (°)	*t*-Value	*p*-Value
HS + TS, pointing	14.54	0.46	0.52	0.604
HS + TS, throwing	16.33	0.76	2.66	0.012 *

HS: *head* straight ahead; TS: *total* straight ahead. * indicates *p* ≤ 0.05.

**Table 2:** Comparison between direct effect (DE) and prismatic power in the dark. One-sided *t*-test against 100 percent (=optical power of the prisms).

Condition	Direct Effect (%)	SE (%)	*t*-Value	*p*-Value
DE, rotated	100	7	0.06	0.478
DE, central	82	7	−2.57	0.008 *

DE: direct effect. Rotated: chair position to the right; Central: central chair position. Averaged values of 28 participants. Results as percentages of prismatic power. * indicates *p* ≤ 0.05.

**Table 3:** The sum of *visual* straight ahead (VS; gaze direction) and *proprioceptive* straight ahead (PS) after prism adaptation vs. aftereffect (AE), in the dark with prisms on. Two-sided paired *t*-test.

Conditions	Mean VS + PS (°)	SE VS + PS (°)	Mean AE (°)	SE AE (°)	*t*-Value	*p*-Value
dark, rotated	−4.80	0.71	−5.07	0.62	0.32	0.752
dark, central	−3.40	0.55	−5.41	0.64	2.68	0.013*

VS: *visual* straight ahead (inverted); PS: *proprioceptive* straight ahead; AE: aftereffect; Rotated: rotated chair position; Central: central chair position. * indicates *p* ≤ 0.05.

**Table 4:** Comparison between direct effects for 30, 60 and 90 pointing movements and prismatic power, before adaptation, in dark and bright laboratory. One-sided *t*-test against 100 percent (=optical power of the prisms).

Conditions	Direct Effect (%)	SE (%)	*t*-Value	*p*-Value
DE, dark 30	86	12	−1.16	0.143
DE, dark 60	86	11	−1.33	0.114
DE, dark 90	83	10	−1.62	0.075 ^T^
DE, light 30	71	9	−3.16	0.008 *
DE, light 60	71	9	−3.18	0.008 *
DE, light 90	72	9	−3.26	0.007 *

DE: direct effect; Dark: adaptation in dark laboratory; Light: adaptation in bright laboratory; for the conditions 30, 60 and 90 movements; Results as percentage of prismatic power. * indicates *p* ≤ 0.05; ^T^ = Trend.

## Supplementary Materials

The supplementary materials about experimental conditions are available online at www.mdpi.com/2411-5150/1/4/27/s1.
