# Supplementary material for: On the Mechanics of Immediate Corrections and Aftereffects in Prism Adaptation
Source: Vision (Basel). 2017 Dec 19;1(4):27. doi: 10.3390/vision1040027 (PMC6836038; doi:10.3390/vision1040027)
Supplement: Supplementary file 1 [file vision-01-00027-s001.pdf]

Supplementary material

Material & Methods

Procedure

Table S1. Experimental conditions.

Block	Condition	Task	No. of PM	No. of P	Target	Arm	Feedback	CP	Prisms	CR	Light
**rotated**	Pretest	HS	-	5	-	-	no	central	-	no	off
		VS	-	5	-	-	no	central	-	yes	off
		PS	5	-	-	right	no	central	-	yes	off
		IT	5	-	-	left	no	central	-	yes	off
	Adaptation	AD	30	-	central	right	yes	right	right	no	off
	1. Posttest	see Pretest									
	Readaptation	READ	30	-	central	right	yes	right	-	no	off
**central**	Pretest	HS	-	5	-	-	no	central	-	no	off
		VS	-	5	-	-	no	central	-	yes	off
		PS	5	-	-	right	no	central	-	yes	off
		IT	5	-	-	left	no	central	-	yes	off
	Adaptation	AD	30	-	central	right	yes	central	right	yes	off
	1. Posttest	see Pretest									
	Readaptation	READ	30	-	central	right	yes	central	-	yes	off

No. of PM: number of pointing movements; No. of P: number of pictures; CP: chair position; CR: chin rest; HS: head straight ahead; VS: visual straight ahead; PS: proprioceptive straight ahead; IT: intermanual transfer; AD: adaptation; READ: readaptation. Grey labeled conditions depict the additional condition in group IT.

Table S2. Experimental conditions.

Block	Condition	Task	No. of PM	No. of P	Target	Feedback	CP	Prisms	CR	Light
**Dark**	Adaptation	AD	30/60/90	-	central	yes	right	right	no	off
	Readaptation	READ	30/60/90	-	central	yes	right	-	no	off
**Light**	Adaptation	AD	30/60/90	-	central	yes	right	right	no	on
	Readaptation	READ	30/60/90	-	central	yes	right	-	no	on

No. of PM: number of pointing movements (30, 60 or 90 movements); No. of P: number of pictures; CP: chair position; CR: chin rest; AD: adaptation; READ: readaptation.
